# SARS-CoV-2 Omicron virus causes attenuated disease in mice and hamsters

**DOI:** 10.1038/s41586-022-04441-6

**Published:** 2022-01-21

**Authors:** Peter J. Halfmann, Shun Iida, Kiyoko Iwatsuki-Horimoto, Tadashi Maemura, Maki Kiso, Suzanne M. Scheaffer, Tamarand L. Darling, Astha Joshi, Samantha Loeber, Gagandeep Singh, Stephanie L. Foster, Baoling Ying, James Brett Case, Zhenlu Chong, Bradley Whitener, Juan Moliva, Katharine Floyd, Michiko Ujie, Noriko Nakajima, Mutsumi Ito, Ryan Wright, Ryuta Uraki, Prajakta Warang, Matthew Gagne, Rong Li, Yuko Sakai-Tagawa, Yanan Liu, Deanna Larson, Jorge E. Osorio, Juan P. Hernandez-Ortiz, Amy R. Henry, Karl Ciuoderis, Kelsey R. Florek, Mit Patel, Abby Odle, Lok-Yin Roy Wong, Allen C. Bateman, Zhongde Wang, Venkata-Viswanadh Edara, Zhenlu Chong, John Franks, Trushar Jeevan, Thomas Fabrizio, Jennifer DeBeauchamp, Lisa Kercher, Patrick Seiler, Ana Silvia Gonzalez-Reiche, Emilia Mia Sordillo, Lauren A. Chang, Harm van Bakel, Viviana Simon, Consortium Mount Sinai Pathogen Surveillance (PSP) study group, B. Alburquerque, H. Alshammary, A. A. Amoako, S. Aslam, R. Banu, C. Cognigni, M. Espinoza-Moraga, K. Farrugia, A. van de Guchte, Z. Khalil, M. Laporte, I. Mena, A. E. Paniz-Mondolfi, J. Polanco, A. Rooker, L. A. Sominsky, Daniel C. Douek, Nancy J. Sullivan, Larissa B. Thackray, Hiroshi Ueki, Seiya Yamayoshi, Masaki Imai, Stanley Perlman, Richard J. Webby, Robert A. Seder, Mehul S. Suthar, Adolfo García-Sastre, Michael Schotsaert, Tadaki Suzuki, Adrianus C. M. Boon, Michael S. Diamond, Yoshihiro Kawaoka

**Affiliations:** 1 Influenza Research Institute, Department of Pathobiological Sciences, School of Veterinary Medicine, University of Wisconsin-Madison, Madison, WI USA; 2 Department of Pathology, National Institute of Infectious Diseases, Tokyo, Japan; 3 Division of Virology, Institute of Medical Science, University of Tokyo, Tokyo, Japan; 4 Department of Medicine, Washington University School of Medicine, St Louis, MO USA; 5 Department of Surgical Sciences, School of Veterinary Medicine, University of Wisconsin–Madison, Madison, WI USA; 6 Department of Microbiology, Icahn School of Medicine at Mount Sinai, New York, NY USA; 7 Global Health and Emerging Pathogens Institute, Icahn School of Medicine at Mount Sinai, New York, NY USA; 8 Center for Childhood Infections and Vaccines of Children’s Healthcare of Atlanta, Department of Pediatrics, Emory Vaccine Center, Emory University School of Medicine, Atlanta, GA USA; 9 Vaccine Research Center, National Institute of Allergy and Infectious Diseases, National Institutes of Health, Bethesda, MD USA; 10 The Research Center for Global Viral Diseases, National Center for Global Health and Medicine Research Institute, Tokyo, Japan; 11 Department of Animal Dairy, and Veterinary Sciences, College of Agriculture and Applied Sciences, Utah State University, Logan, UT USA; 12 Department of Pathobiological Sciences, School of Veterinary Medicine, University of Wisconsin, Madison, WI USA; 13 Colombia/Wisconsin One-Health Consortium and One-Health Genomic Laboratory, Universidad Nacional de Colombia, Medellín, Colombia; 14 Wisconsin State Laboratory of Hygiene, Madison, WI USA; 15 Department of Microbiology and Immunology, University of Iowa, Iowa City, IA USA; 16 Department of Infectious Diseases, St Jude Children’s Research Hospital, Memphis, Tennessee USA; 17 Department of Genetics and Genomic Sciences, Icahn School of Medicine at Mount Sinai, New York, NY USA; 18 Department of Pathology, Molecular and Cell-Based Medicine, Icahn School of Medicine at Mount Sinai, New York, NY USA; 19 Graduate School of Biomedical Sciences, Icahn School of Medicine at Mount Sinai, New York, NY USA; 20 Department of Medicine, Division of Infectious Diseases, Icahn School of Medicine at Mount Sinai, New York, NY USA; 21 Department of Microbiology and Immunology, Emory University, Atlanta, GA USA; 22 The Tisch Cancer Institute, Icahn School of Medicine at Mount Sinai, New York, NY USA; 23 Department of Pathology & Immunology, Washington University School of Medicine, St Louis, MO USA; 24 Department of Molecular Microbiology, Washington University School of Medicine, St Louis, MO USA; 25 The Andrew M. and Jane M. Bursky Center for Human Immunology and Immunotherapy Programs, Washington University School of Medicine, St Louis, MO USA

**Keywords:** Pathogens, SARS-CoV-2

## Abstract

The recent emergence of B.1.1.529, the Omicron variant^1,2^, has raised concerns of escape from protection by vaccines and therapeutic antibodies. A key test for potential countermeasures against B.1.1.529 is their activity in preclinical rodent models of respiratory tract disease. Here, using the collaborative network of the SARS-CoV-2 Assessment of Viral Evolution (SAVE) programme of the National Institute of Allergy and Infectious Diseases (NIAID), we evaluated the ability of several B.1.1.529 isolates to cause infection and disease in immunocompetent and human ACE2 (hACE2)-expressing mice and hamsters. Despite modelling data indicating that B.1.1.529 spike can bind more avidly to mouse ACE2 (refs. ^3,4^), we observed less infection by B.1.1.529 in 129, C57BL/6, BALB/c and K18-hACE2 transgenic mice than by previous SARS-CoV-2 variants, with limited weight loss and lower viral burden in the upper and lower respiratory tracts. In wild-type and hACE2 transgenic hamsters, lung infection, clinical disease and pathology with B.1.1.529 were also milder than with historical isolates or other SARS-CoV-2 variants of concern. Overall, experiments from the SAVE/NIAID network with several B.1.1.529 isolates demonstrate attenuated lung disease in rodents, which parallels preliminary human clinical data.

## Abstract (web summary)

A collaborative study demonstrates that, compared with previous SARS-CoV-2 variants, B.1.1.529 isolates cause less infection and disease in mice and hamsters, in agreement with preliminary data from studies in humans.

## Main

Severe acute respiratory syndrome coronavirus 2 (SARS-CoV-2) has caused a pandemic resulting in millions of deaths worldwide. The extensive morbidity and mortality made the development of vaccines, antibody-based countermeasures and antiviral agents a global health priority. As part of this process, several models of SARS-CoV-2 infection and lung pathogenesis were developed in animals for rapid testing^5^. Remarkably, several highly effective vaccines and therapeutics were deployed with billions of doses given worldwide. Although these measures markedly reduced hospitalizations and deaths, their efficacy has been jeopardized by the emergence of SARS-CoV-2 variants with mutations in the spike gene.

The SARS-CoV-2 spike protein engages angiotensin-converting enzyme 2 (ACE2) on the surface of human cells to facilitate entry and infection of cells^6^. Upon cell attachment, spike proteins are cleaved by host proteases into S1 and S2 fragments. The S1 protein includes the amino-terminal (NTD) and receptor-binding (RBD) domains. The RBD is the target of many potently neutralizing monoclonal^7–11^ and serum polyclonal^12^ antibodies. Although SARS-CoV-2 spike proteins from strains early in the pandemic bound to ACE2 from several animal species (for example, hamster, ferret and nonhuman primates), they did not bind mouse ACE2, which explained why laboratory strains of mice could not be infected by SARS-CoV-2 (refs. ^6,13^); indeed, mice could become susceptible through expression of hACE2 via a transgene^14–16^ or viral vector^17,18^, or under regulation of the mouse ACE2 promoter^19–21^. Later in the pandemic, several strains acquired a mouse-adapting spike substitution (N501Y), which allowed engagement of mouse ACE2 and productive infection of mice without hACE2 expression^22–24^.

In late November 2021, the Omicron (B.1.1.529) variant emerged. This variant has the largest number (>30) of substitutions, deletions or insertions in the spike protein described so far, raising concerns of escape from protection by vaccines and therapeutic monoclonal antibodies. B.1.1.529 isolates have many changes in the RBD (G339D, R346K, S371L, S373P, S375F, K417N, N440K, G446S, S477N, T478K, E484A, Q493R, G496S, Q498R, N501Y and Y505H). The N501Y substitution along with changes at sites (K417, E484, Q493, Q498 and N501) associated with mouse adaptation^25–30^ indicated that B.1.1.529 should infect mice^3^. One study speculated that the progenitor of B.1.1.529 jumped from humans to mice, and then back into humans^4^. In support of this, B.1.1.529 RBD binds to mouse ACE2 (ref. ^31^). Last, hamsters have been a valuable animal model for assessing countermeasures against SARS-CoV-2 and variants. Hamsters are susceptible to SARS-CoV-2 infection and show similar pathological changes to those seen in lung tissues from COVID-19 patients^5,32,33^. Here, using data from several laboratories of the SAVE/NIAID consortium (Supplementary Table 1), we report on the infectivity of several B.1.1.529 isolates in mice and hamsters, two key rodent models of SARS-CoV-2 infection and pathogenesis.

## B.1.1.529 infection in mice

**Fig. 1:**
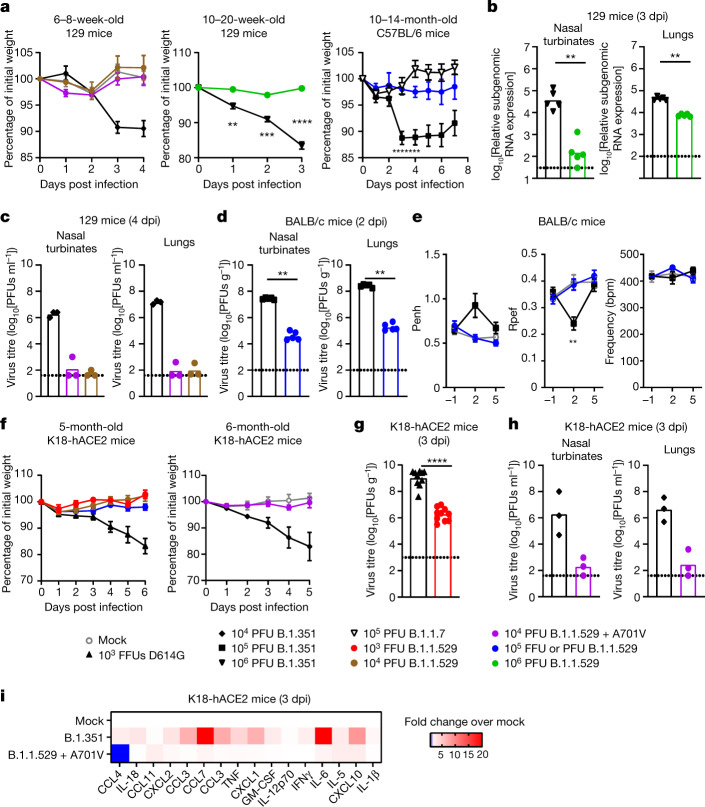
B.1.1.529 is less pathogenic in mice. **a**, Left: weight change in mock-infected mice (*n* = 4) or mice inoculated with B.1.1.529 + A701V (*n* = 5), B.1.1.529 (*n* = 3) or B.1.351 (*n* = 3). Middle: weight change in mice inoculated with B.1.1.529 or B.1.351 (*n* = 5) (**P = 0.0075, ***P = 0.0006, ****P < 0.0001). Right: weight change in mice inoculated with B.1.1.529 (*n* = 4), B.1.1.7 (*n* = 10) or B.1.351 (*n* = 18). Comparison between B.1.351 and B.1.1.529: *P = 0.0151, ***P = 0.0003 (3 dpi) and 0.0006 (4 dpi). Mean ± s.e.m. **b**, Viral RNA level in mice inoculated with B.1.1.529 or B.1.351 (*n* = 5) (**P = 0.0079). **c**, Infectious virus titre in mice inoculated with B.1.1.529 + A701V, B.1.1.529 or B.1.351 (*n* = 3). **d**, Infectious virus titre in mice inoculated with B.1.1.529 or B.1.351 (*n* = 5) (**P = 0.0079). **e**, Pulmonary function analysis as measured by whole-body plethysmography. Mean ± s.e.m. Comparison between B.1.617.2 and B.1.351: **P = 0.0095 (*n* = 5 each). **f**, Left, weight change in mice inoculated with WA1/2020 D614G (10^3^ FFU; *n* = 6), B.1.1.529 (10^3^ FFU; *n* = 3), B.1.1.529 (10^4^ PFU; *n* = 6) or B.1.1.529 (10^5^ FFU; *n* = 3). Right, weight change in mice inoculated with 10^4^ PFU of B.1.1.529 +A701V (*n* = 6) or B.1.351 (*n* = 6), or mock-infected, age-matched mice (*n* = 4). Mean ± s.e.m. **g**, Infectious virus titre in lungs of mice inoculated with WA1/2020 D614G (*n* = 8) or B.1.1.529 (*n* = 7) (****P < 0.0001). **h**, Infectious virus titre in mice inoculated with B.1.1.529 + A701V or B.1.351 (*n* = 3). **i**, Heat map of concentration of cytokines and chemokines in lungs of infected mice. Results are from one (**a**–**f**, **h**, **i**) or two (**g**) experiments. The dotted line is the limit of detection. Statistical analysis (**a**, **e**: two-way analysis of variance (ANOVA) with multiple comparisons test; **b**, **d**, **g**: two-tailed Mann–Whitney test) was performed on datasets with four or more data points. See Supplementary Table 1 for more information. CCL4, chemokine (C-C motif) ligand 4; IL-18, interleukin-18; CXCL2, chemokine (C-X-C motif) ligand 2; TNF, tumour necrosis factor; GM-CSF, granulocyte–macrophage CSF; IFNγ, interferon-γ. Source data

Because of the presence of several amino acid alterations that are considered mouse adapting, we predicted that B.1.1.529 should infect immunocompetent mice and cause lung disease as seen with other recombinant strains (WA1/2020 N501Y) or variants (for example, B.1.351) containing N501Y substitutions. We first tested B.1.1.529 in 129 mice. Two of our laboratories independently inoculated 6–8-week-old or 10–20-week-old 129 mice with 10^4^, 10^5^ or 10^6^ infectious units (plaque-forming units (PFU) or focus-forming units (FFU)) of three different B.1.1.529 strains (Supplementary Tables 1 and 2). As 129 mice sustain 10 to 15% loss of body weight 3 to 4 days post infection (dpi) yet recover and gain weight beginning at 5 dpi (refs. ^22,34^) with SARS-CoV-2 strains encoding N501Y substitutions^34^, we assessed weight change with B.1.1.529 at 3 and 4 dpi. However, after inoculation with B.1.1.529, 129 mice failed to lose weight (Fig. 1a). Similarly, aged (10- to 14-month-old) C57BL/6 mice also did not lose weight after B.1.1.529 infection, whereas those infected with B.1.351 did (Fig. 1a).

We next compared viral burden in B.1.1.529- and B.1.351-infected 129 mice. At 3 dpi, 129 mice infected with B.1.351 sustained high levels of infection in the nasal wash, nasal turbinates and lungs (Fig. 1b). The levels of viral RNA in the nasal turbinates and lungs of B.1.1.529-infected mice were 10- to 100-fold lower than those in B.1.351-infected animals (Fig. 1b). Similar results were seen in a separate cohort of 129 mice at 4 dpi, with 1,000- to 100,000-fold less infectious virus recovered from nasal turbinates and lungs of animals infected with B.1.1.529 compared to B.1.351 (Fig. 1c).

Members of the group also tested B.1.1.529 in BALB/c mice. At 2 dpi, infectious virus levels in the nasal turbinates and lungs were significantly lower (≈1,000-fold, *P* < 0.001) in BALB/c mice infected with B.1.1.529 compared to B.1.351 (Fig. 1d). We used whole-body plethysmography^35^ to measure pulmonary function in infected mice. At 2 dpi, whereas B.1.351 caused an increase (*P* < 0.001) in the lung enhanced pause (Penh), a marker of bronchoconstriction, B.1.1.529 did not (Fig. 1e). The ratio of peak expiratory flow (Rpef) also was decreased at 2 dpi in BALB/c mice infected with B.1.351 but not B.1.1.529 (*P* < 0.001, Fig. 1e).

Two of our groups tested B.1.1.529 infection in K18-hACE2 transgenic mice, which express hACE2 under an epithelial cytokeratin promoter^14^, and are more susceptible to SARS-CoV-2 infection^16^. At intranasal doses ranging from 10^3^ to 10^5^ infectious units of B.1.1.529, weight loss was not observed over the first 5 to 6 days of infection in younger or older K18-hACE2 mice (Fig. 1f). These data contrast with historical results with WA1/2020 D614G or variant (for example, B.1.351) SARS-CoV-2 strains^16,24,34,36^, which uniformly induce weight loss starting at 4 dpi. The groups separately observed reduced levels of infectious B.1.1.529 compared to WA1/2020 D614G or B.1.351 in the lower respiratory tracts at 3 dpi (Fig. 1g, h). Finally, we assessed inflammatory responses in the lungs of K18-hACE2 mice at 3 dpi. Mice inoculated with B.1.1.529 had lower levels of several pro-inflammatory cytokines and chemokines compared to those inoculated with B.1.351, with many values similar to those of uninfected controls (Fig. 1i and Supplementary Table 3). Thus, on the basis of several parameters (weight change, viral burden, respiratory function measurements and cytokine responses), B.1.1.529 seems attenuated in the respiratory tract of several strains of mice.

## B.1.1.529 infection in hamsters

**Fig. 2:**
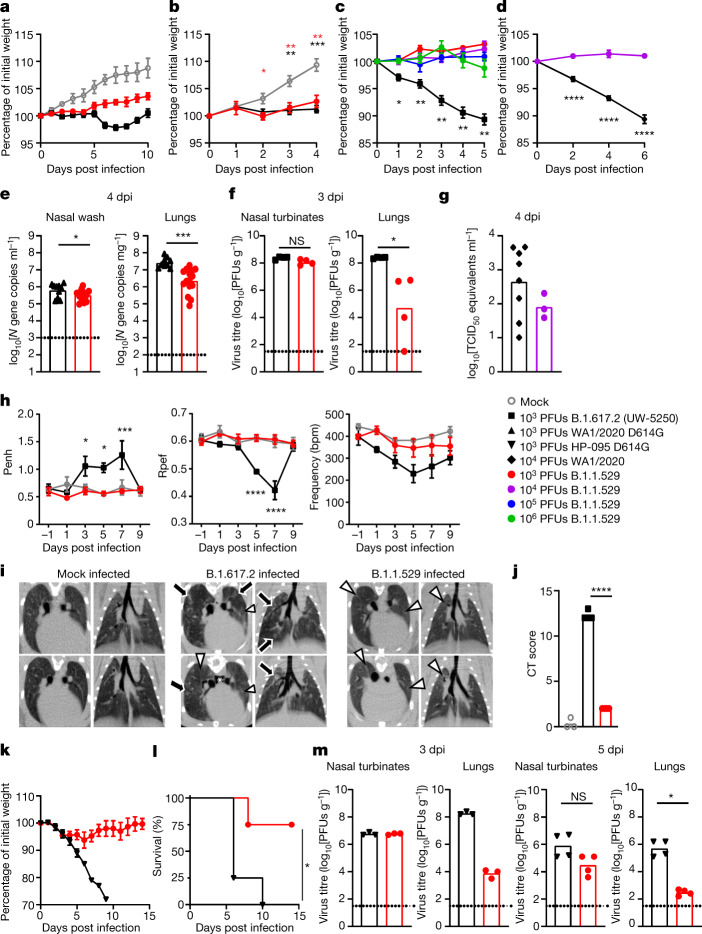
B.1.1.529 is less pathogenic in wild-type and hACE2-transgenic Syrian hamsters. **a**, Weight change in uninfected age-matched hamsters (*n* = 3) or in hamsters inoculated with B.1.1.529 or B.1.617.2 (*n* = 4). Mean ± s.e.m. **b**, Weight change in uninfected age-matched hamsters (*n* = 9) or in hamsters inoculated with B.1.1.529 (*n* = 10) or WA1/2020 D614G (*n* = 6). Mean ± s.e.m. (red, *P = 0.0293; red, **P = 0.0046 and 0.0014; black, **P = 0.0021; black, ***P = 0.0001). **c**, Weight change in hamsters inoculated with 10^3^, 10^4^, 10^5^ or 10^6^ PFU of B.1.1.529 or 10^3^ PFU of B.1.617.2 (*n* = 4). Mean ± s.e.m. Comparison between B.1.617.2 and B.1.1.529 (10^3^ PFU): *P = 0.0476, **P = 0.0041, 0.0041, 0.0047 and 0.0019, respectively. **d**, Weight change in hamsters inoculated with B.1.1.529 (*n* = 5) or WA1/2020 (*n* = 9). Mean ± s.e.m. (****P < 0.0001). **e**, Viral RNA level in hamsters inoculated with WA1/2020 D614G or B.1.1.529 (*n* = 15) (*P = 0.015, ***P < 0.0003). **f**, Infectious virus titre in hamsters inoculated with B.1.617.2 or B.1.1.529 (*n* = 4) (*P = 0.0286; NS, not significant). **g**, Nasal wash viral RNA level in hamsters inoculated with WA1/2020 (*n* = 8) or B.1.1.529 (*n* = 3). TCID_50_, median tissue culture infectious dose. **h**, Pulmonary function analysis by whole-body plethysmography. Mean ± s.e.m. (Penh and Rpef, comparison between B.1.617.2 and B.1.1.529: *P = 0.0263 (3 dpi), *P = 0.0186 (5 dpi), ***P = 0.0005 (7 dpi), ****P < 0.0001) (*n* = 4). **i**, Micro-CT images of the lungs of mock-infected (*n* = 3) or B.1.617.2- (*n* = 4) and B.1.1.529-infected (*n* = 4) hamsters at 7 dpi. Multifocal nodules (black arrows), ground-glass opacity (white arrowheads) and pneumomediastinum (white asterisk) are indicated. **j**, CT score for uninfected hamsters (*n* = 3) or those inoculated with B.1.617.2 or B.1.1.529 (*n* = 4) (****P < 0.0001). **k**, Weight change in hACE2 hamsters inoculated with HP-095 D614G or B.1.1.529 (*n* = 4). Error bars indicate s.e.m. **l**, Survival of hACE2 hamsters after inoculation with HP-095 D614G or B.1.1.529 (*n* = 4) (*P = 0.029). **m**, Infectious virus titre of hACE2 hamsters inoculated with HP-095 D614G or B.1.1.529; *n* = 3 (3 dpi), *n* = 4 (5 dpi) (*P = 0.0286). The results are from one (**a**, **c**, **d**, **f**–**m**) or two to three independent (**b**, **e**) experiments. Dotted lines represent the limit of detection. Statistical analysis (**b**–**d**, **h**: two-way ANOVA with multiple comparisons test; **e**, **j**: two-tailed *t*-test, **f**, **m**: two-tailed Mann–Whitney test, **l**: log-rank test) was performed on datasets with four or more data points. See Supplementary Table 1 for more information. Source data

Four members of our group tested three different B.1.1.529 strains for their ability to infect and cause disease (Supplementary Table 1). Whereas intranasal infection with historical or other variant SARS-CoV-2 strains generally resulted in ≈10 to 15% reduction in body weight over the first week, we observed no weight loss in hamsters inoculated with B.1.1.529 (Fig. 2a–d), although animals did not gain body weight as rapidly as uninfected hamsters. Viral RNA analysis at 4 dpi showed lower levels of B.1.1.529 infection in the lungs (12-fold, *P* < 0.001) compared to WA1/2020 D614G (Fig. 2e). A comparison of infectious viral burden in tissues at 3 dpi between B.1.617.2 and B.1.1.529 strains showed virtually no difference in nasal turbinates but substantially less infection of B.1.1.529 in the lungs of most animals (Fig. 2f). A comparison of viral RNA levels between WA1/2020 and B.1.1.529 in nasal washes at 4 dpi did not show substantial differences in titres (Fig. 2g). Thus, in hamsters infected with B.1.1.529, the upper, but not the lower, respiratory tract infection seems relatively intact.

We used whole-body plethysmography to measure pulmonary function in infected Syrian hamsters. Starting at 3 dpi and continuing until 7 dpi, infection with B.1.617.2 caused an increase (*P* < 0.05) in the Penh, whereas B.1.1.529 infection did not (Fig. 2h, left). The Rpef was decreased at 5 and 7 dpi in animals infected with B.1.617.2 but not B.1.1.529 (*P* < 0.001; Fig. 2h, middle). Finally, hamsters infected with B.1.617.2, but not B.1.1.529, demonstrated a decrease in respiratory rate (frequency) compared to uninfected animals (Fig. 2h, right). On the basis of several functional parameters, lung infection and disease after B.1.1.529 infection was attenuated compared to that after infection with other variant strains.

We performed microcomputed tomography (micro-CT) to assess for lung abnormalities in hamsters at 7 dpi. Micro-CT analysis revealed lung abnormalities in all B.1.617.2-infected hamsters on 7 dpi that were consistent with commonly reported imaging features of COVID-19 pneumonia^37^. In comparison, analysis of B.1.1.529-infected hamsters on 7 dpi revealed patchy, ill-defined ground-glass opacity consistent with minimal to mild pneumonia. Syrian hamsters infected with B.1.617.2 had a much higher CT disease score^35^ than those infected with B.1.1.529 (Fig. 2i, j).

**Fig. 3:**
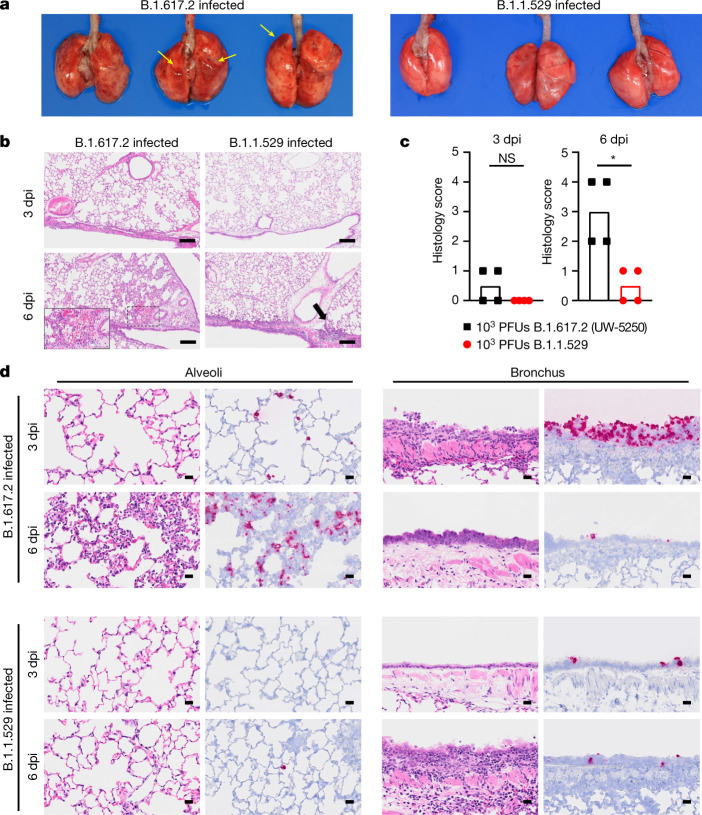
Pathological findings in the lungs of SARS-CoV-2-infected Syrian hamsters. Hamsters were inoculated with 10^3^ PFU of B.1.617.2 or B.1.1.529 and euthanized at 3 and 6 dpi (*n* = 4). **a**, Macroscopic images of the lungs obtained at 6 dpi. Yellow arrows indicate haemorrhage. **b**, Lung sections from animals infected with B.1.617.2 or B.1.1.529. Scale bars, 200 µm. Focal alveolar haemorrhage in B.1.617.2-infected animals at 6 dpi is outlined and shown at higher magnification in the inset (scale bar, 100 µm). Black arrow indicates focal inflammation. **c**, Histopathological score of pneumonia based on the percentage of alveolitis in a given section using the following scoring: 0, no pathological change; 1, affected area (≤10%); 2, affected area (<50%, >10%); 3, affected area (≥50%); an additional point was added when pulmonary edema and/or alveolar haemorrhage was observed. Data are median score (*n* = 4; *P = 0.0286; two-tailed Mann–Whitney test). **d**, RNA in situ hybridization for SARS-CoV-2 viral RNA. Representative images for the alveoli and bronchi of hamsters infected with B.1.617.2 or B.1.1.529 (*n* = 4) virus at 3 or 6 dpi are shown. Scale bars, 20 µm. See Supplementary Table 1 for more information. Source data

Members of our group also compared lung pathology in Syrian hamsters after infection with B.1.617.2 or B.1.1.529. The lungs obtained from B.1.617.2-infected hamsters showed congestion and/or haemorrhage, which were absent in B.1.1.529-infected animals (Fig. 3a). Immune cell infiltration and inflammation were present in the peribronchial regions of the lungs at 3 dpi with B.1.617.2. At 6 dpi, extensive infiltration of neutrophils and lymphocytes in the alveolar space was accompanied by pulmonary edema and haemorrhage (Fig. 3b, inset), and regenerative changes in the bronchial epithelia became prominent (Fig. 3b). By contrast, in B.1.1.529-infected hamsters, small foci of inflammation in the alveoli and peribronchial regions were observed only at 6 dpi (Fig. 3b). A worse histopathology score of viral pneumonia at 6 dpi was measured after B.1.617.2 than B.1.1.529 infection (Fig. 3c). After B.1.617.2 infection, viral RNA was detected readily in the alveoli and bronchial epithelia at 3 and 6 dpi (Fig. 3d). After B.1.1.529 infection, fewer bronchial epithelial cells and alveoli were positive for viral RNA at either time point (Fig. 3d). Thus, B.1.1.529 replicates less efficiently in the lungs of Syrian hamsters, which results in less severe pneumonia compared to that resulting from the B.1.617.2 variant.

Although hamster ACE2 can serve as a receptor for the SARS-CoV-2 spike protein, some of the contact residues in hACE2 are not conserved^38^, which could diminish infectivity. To develop a more susceptible hamster model, members of the consortium used transgenic hamsters expressing hACE2 under the epithelial cytokeratin-18 promoter^39^. Whereas intranasal inoculation of 10^3^ PFU of HP-095 D614G virus resulted in marked weight loss within the first week (Fig. 2k) and uniform mortality by 10 dpi (Fig. 2l), less weight loss and death (*P* < 0.05) were observed after infection with B.1.1.529. Moreover, 1,000- to 10,000-fold lower levels of infectious virus were measured in the lungs of hACE2 transgenic hamsters challenged with B.1.1.529 than in those challenged with HP-095 D614G at 3 and 5 dpi (Fig. 2m). As seen in wild-type Syrian hamsters, smaller differences in infection were observed in the nasal turbinates. Thus, B.1.1.529 infection in the lung is attenuated in both wild-type and hACE2 transgenic hamsters.

## Discussion

Our experiments indicate that the B.1.1.529 variant is less pathogenic in laboratory mice and hamsters. Although these results are consistent with preliminary data in humans^40,41^, the basis for attenuation remains unknown. One study indicates that B.1.1.529 replicates faster in the human bronchus and less in lung cells, which may explain its greater transmissibility and putative lower disease severity^42^. We observed that B.1.1.529 resulted in a lower level of infection of hamster bronchial cells in vivo and lower viral burden in nasal washes and turbinates in mice compared with other SARS-CoV-2 strains. The attenuation in mice was unexpected given that B.1.1.529 has alterations in the RBD that are sites associated with adaptation for mice^25–27^. The attenuation in hamsters seen by our group and others^43^ was also surprising, given that other SARS-CoV-2 variants replicate to high levels in this animal^35,44,45^. Whereas the >30 changes in the B.1.1.529 spike protein could impact receptor engagement, changes in other proteins could affect replication, temperature sensitivity, cell and tissue tropism, and induction of pro-inflammatory responses in a species-specific manner. Our results showing attenuated B.1.1.529 infection in laboratory mice do not support the suggestion that B.1.1.529 has a mouse origin^4^. However, infection studies in wild mice^46^ are needed to fully address this question.

Although B.1.1.529 is less pathogenic in mice and hamsters, these animals still will have utility in evaluating vaccine, antibody or small-molecule inhibitors. The mice and hamsters tested, to varying degrees, showed evidence of viral replication and dissemination to the lower respiratory tract, which could be mitigated by countermeasures. The most severe B.1.1.529 infection and disease was observed in hACE2-expressing mice and hamsters, which is consistent with findings for other SARS-CoV-2 strains^16,24,39,47^, and possibly related to the enhanced interactions between hACE2 and B.1.1.529 spike^48^. Indeed, structural analysis of the B.1.1.529 spike protein in complex with hACE2 reveals new interactions formed by mutated residues in the RBD^48,49^.

These in vivo studies were performed as part of the SAVE/NIAID consortium and reflect a network that communicates weekly to expedite progress on SARS-CoV-2 variants. This format had several advantages: animal experiments were reproduced across laboratories providing confidence in results; several B.1.1.529 isolates were tested limiting the possibility of sequence adaptations in a strain from one laboratory that could skew results; several strains of mice and hamsters at different ages were tested allowing for a comprehensive dataset; and the groups used overlapping metrics to evaluate infection and disease in the different animal models.

We note several limitations to our study. First, our experiments reflect data from a consortium that did not use uniform study design and metrics, which created variability in outcomes; despite this, data from several groups indicate that B.1.1.529 is attenuated in rodent models. Second, although attenuation of B.1.1.529 in mice and hamsters correlates with preliminary data in humans, evaluation in nonhuman primates and unvaccinated, previously uninfected humans is needed for corroboration. Third, we used the prevailing B.1.1.529 isolate that lacks an R346K substitution. Approximately 10% of B.1.1.529 sequences in GISAID as of the writing of this paper have an R346K sequence, and this substitution or others in gene products apart from spike might affect virulence. Although one of the B.1.1.529 isolates we tested contains an additional A701V change in spike near the furin cleavage site, it was still attenuated in mice. Fourth, detailed pathological and immunological analyses were not performed for all of the animal species studied. It remains possible that B.1.1.529 is attenuated clinically (for example, weight loss) because of defects in promoting pathological host responses.

In summary, our collective studies rapidly and reproducibly demonstrated attenuated infection in several strains of mice and hamsters. Experiments are ongoing to determine the basis for attenuation in mice and hamsters and to determine how this relates to B.1.1.529 infection in humans.

## Methods

### Cells

Vero-TMPRSS2 (refs. ^35,50,51^) and Vero-hACE2-TMPRSS2 (ref. ^52^) cells were cultured at 37 °C in Dulbecco’s modified Eagle medium (DMEM) supplemented with 10% fetal bovine serum (FBS), 10 mM HEPES pH 7.3 and 100 U ml^−1^ penicillin–streptomycin. Vero-TMPRSS2 cells were supplemented with 5 μg ml^−1^ blasticidin or 1 mg ml^−1^ geneticin (depending on the cell line) and in some cultures with Plasmocin. Vero-hACE2-TMPRSS2 cells were supplemented with 10 µg ml^−1^ puromycin. All cells routinely tested negative for mycoplasma using a PCR-based assay.

### Viruses

The WA1/2020 recombinant strains with substitutions (D614G and/or N501Y/D614G) were described previously^53^. The B.1.1.529 isolates (hCoV-19/USA/WI-WSLH-221686/2021 (GISAID: EPI_ISL_7263803), hCoV-19/Japan/NC928-2N/2021 (NC928) (GISAID: EPI_ISL_7507055), hCoV-19/USA/NY-MSHSPSP-PV44476/2021 (GISAID: EPI_ISL_7908052), hCoV-19/USA/NY-MSHSPSP-PV44488/2021 (GISAID: EPI_ISL_7908059) and hCoV-19/USA/GA-EHC-2811C/2021 (GISAID: EPI_ISL_7171744)) were obtained from nasal swabs and passaged on Vero-TMPRSS2 cells as described previously^33,35,51^. Sequence differences between B.1.1.529 isolates are depicted in Supplementary Table 2. Other viruses used included: SARS-CoV-2/UT-HP095-1N/Human/2020/Tokyo (HP-095; D614G), hCoV-19/USA/CA_CDC_5574/2020 (Alpha, B.1.1.7; BEI NR54011), hCoV-19/USA/MD-HP01542/2021 (Beta, B.1.351), 20H/501Y.V2 (Beta, B.1.351), hCoV-19/USA/PHC658/202 (Delta, B.1.617.2) and hCoV-19/USA/WI-UW-5250/2021 (Delta, B.1.617.2; UW-5250)^54^. All viruses were subjected to next-generation sequencing as described previously^55^ to confirm the stability of substitutions and avoid introduction of adventitious alterations All virus experiments were performed in an approved biosafety level 3 facility.

### Animal experiments and approvals

Animal studies were carried out in accordance with the recommendations in the Guide for the Care and Use of Laboratory Animals of the National Institutes of Health. The protocols were approved by the Institutional Animal Care and Use Committee at the Washington University School of Medicine (assurance number A3381–01), University of Wisconsin, Madison (V006426), St Jude Children’s Research Hospital (assurance number D16-00043), Emory University, University of Iowa (assurance number A3021-01), Icahn School of Medicine at Mount Sinai (PROTO202100007), BIOQUAL, Inc., and the Animal Experiment Committee of the Institute of Medical Science, the University of Tokyo (approval numbers PA19-72 and PA19-75). Virus inoculations were performed under anaesthesia that was induced and maintained with ketamine hydrochloride and xylazine, and all efforts were made to minimize animal suffering. In vivo studies were not blinded, and animals were randomly assigned to infection groups. No sample-size calculations were performed to power each study. Instead, sample sizes were determined based on previous in vivo virus challenge experiments.

### Mouse infection experiments

Heterozygous K18-hACE2 C57BL/6J mice (strain 2B6.Cg-Tg(K18-ACE2)2Prlmn/J), 129 mice (strain 129S2/SvPasCrl or 129S1/SvImJ) and C57BL/6 (strain 000664) mice were obtained from The Jackson Laboratory and Charles River Laboratories. BALB/c mice were purchased from Japan SLC Inc. Animals were housed in groups and fed standard chow diets. Infection experiments were performed as follows. In a first set of experiments, 5-month-old female K18-hACE2 mice were inoculated intranasally with 10^3^, 10^4^ or 10^5^ FFU of SARS-CoV-2. In a second set of experiments, 129S1 male and female mice were used between 10 and 20 weeks of age. Mice were anaesthetized with isoflurane and inoculated intranasally with virus (50 μl, 10^6^ PFU per mouse). In a third set of experiments, 6-week-old female BALB/c mice were inoculated intranasally with 10^5^ PFU of hCoV-19/Japan/NC928-2N/2021 or hCoV-19/USA/MD-HP01542/2021. In a fourth set of experiments, retired breeder female C57BL/6 mice (10 to 14 months old) were anaesthetized with ketamine–xylazine and inoculated intranasally with SARS-CoV-2 in a total volume of 50 μl DMEM. Animal weight and health were monitored daily. In a fifth set of experiments, 6–8-week-old female 129S1 mice and 6-month-old female K18-hACE2 mice were inoculated intranasally under light ketamine–xylazine sedation with 10^4^ PFU of hCoV-19/USA/NY-MSHSPSP-PV44476/2021 or hCoV-19/USA/NY-MSHSPSP-PV44488/2021 in a total volume of 50 μl.

### Hamster infection experiments

Male 5–6-week-old Syrian golden hamsters were obtained from Charles River Laboratories, Envigo or Japan SLC Inc. The K18-hACE2 transgenic hamster line was developed with a piggyBac-mediated transgenic approach, in which the K18-hACE2 cassette from the pK18-hACE2 plasmid^14^ was transferred into a piggyBac vector, pmhyGENIE-3 (ref. ^56^), for pronuclear injection. hACE2 transgenic hamsters will be described in detail elsewhere^39^. Female 12-month-old transgenic animals were used. Infection experiments were performed as follows. In a first set of experiments, animals were challenged intranasally with 10^3^ PFU of WA1/2020 D614G or B.1.1.529 variant in 100 µl. In a second set of experiments, under isoflurane anaesthesia, wild-type Syrian hamsters were intranasally inoculated with 10^3^ PFU of SARS-CoV-2 strains in 30 µl. Body weight was monitored daily. For virological and pathological examinations, four hamsters per group were euthanized at 3 and 6 dpi, and nasal turbinates and lungs were collected. The virus titres in the nasal turbinates and lungs were determined by plaque assays on Vero-TMPRSS2 cells. Human ACE2 transgenic hamsters were intranasally inoculated with 10^3^ PFU of HP-095 D614G or B.1.1.529 (hCoV-19/USA/WI-WSLH-221686/2021) in 50 µl. Body weight and survival were monitored daily, and nasal turbinates and lungs were collected at 3 and 5 dpi for virological analysis. In a third set of experiments, six-week-old male Syrian golden hamsters were randomized into groups of 4 to 6 and inoculated with SARS-CoV-2 via delivery of 100 µl of appropriately diluted virus in PBS equally split between both nostrils. Weight change and clinical observations were collected daily. In a fourth set of experiments, while under isoflurane anaesthesia, male 8–10-week-old hamsters were inoculated intranasally with 10^4^ PFU of WA1/2020 or B.1.1.529 in 100 µl volume. Body weight and survival were monitored daily. Nasal washes were taken at 4 dpi for virological analysis.

### Measurement of viral burden

#### Mouse studies

Tissues were weighed and homogenized with zirconia beads in a MagNA Lyser instrument (Roche Life Science) in 1,000 μl DMEM medium supplemented with 2% heat-inactivated FBS. Tissue homogenates were clarified by centrifugation at 10,000 r.p.m. for 5 min and stored at −80 °C. RNA was extracted using the MagMax mirVana Total RNA isolation kit (Thermo Fisher Scientific) on the Kingfisher Flex extraction robot (Thermo Fisher Scientific). Viral RNA (*N* gene) was reverse transcribed and amplified using the TaqMan RNA-to-CT 1-Step Kit (Thermo Fisher Scientific), and data were analysed and normalized as described previously^57^. Infectious virus titres were determined by plaque assay on Vero-hACE2-TMPRSS2 cells as previously published^24^. The viral titres in the nasal turbinates and lungs were determined by plaque assay on Vero-TMPRSS2 cells as previously published^51^. At the indicated day post infection, mice were euthanized with isoflurane overdose and one lobe of lung tissue was collected in an Omni Bead ruptor tube filled with Tri Reagent (Zymo, number R2050-1-200). Tissue was homogenized using an Omni Bead Ruptor 24 (5.15 ms, 15 s), and then centrifuged to remove debris. RNA was extracted using a Direct-zol RNA MiniPrep Kit (Zymo, number R2051), and then converted to cDNA using a High-capacity Reverse Transcriptase cDNA Kit (Thermo, number 4368813). SARS-CoV-2 RNA-dependent RNA polymerase and subgenomic RNA were measured as described previously^29,58^. The subgenomic SARS-CoV-2 RNA levels were quantified in nasal turbinates and lungs by quantitative PCR with reverse transcription as previously published^29,55^. Infectious virus titres in nasal turbinates and lungs were determined by plaque assay on Vero-TMPRSS2 cells as described previously^59^.

#### Hamster studies

Lungs were collected 4 dpi and homogenized in 1.0 ml of DMEM, clarified by centrifugation (1,000 *g* for 5 min) and stored at −80 °C. Nasal washes were clarified by centrifugation (2,000 *g* for 10 min) and the supernatant was stored at −80 °C. To quantify viral load in lung tissue homogenates and nasal washes, RNA was extracted from 100 µl samples using E.Z.N.A. Total RNA Kit I (Omega) and eluted with 50 µl of water. Four microlitres of RNA was used for real-time quantitative PCR with reverse transcription to detect and quantify the *N* gene of SARS-CoV-2 using TaqMan RNA-to-CT 1-Step Kit (Thermo Fisher Scientific) as described previously^60^. The virus titres in the nasal turbinates and lungs were determined by plaque assay on Vero E6 cells expressing human TMRPSS2 as previously published^61^. RNA was extracted from clarified nasal washes using the Qiagen RNeasy extraction kit (Qiagen) following the manufacturer’s instructions. Samples were purified on the included columns and eluted in 50 µl of nuclease-free water. PCR was conducted using 4× TaqMan Fast Virus Master Mix (Thermo Fisher) and an *N*-gene primer/probe set.

### Plaque assay

Vero-TMPRSS2 or Vero-TMPRSS2-hACE2 cells were seeded at a density of 1 × 10^5^ cells per well in 24-well tissue culture plates. The following day, medium was removed and replaced with 200 μl of material to be titrated diluted serially in DMEM supplemented with 2% FBS. One hour later, 1 ml of methylcellulose overlay was added. Plates were incubated for 72 h, and then fixed with 4% paraformaldehyde (final concentration) in PBS for 20 min. Plates were stained with 0.05% (w/v) crystal violet in 20% methanol and washed twice with distilled, deionized water.

### Measurement of cytokines and chemokines

Superior and middle lobes of the lungs from K18-hACE2 mice (mock-infected or 3 dpi) were collected, homogenized and then stored at −80 °C. After thawing, lung homogenates were centrifuged at 10,000*g* for 5 min at 4 °C. Samples were inactivated with ultraviolent light in a clear, U-bottom 96-well plate (Falcon). A mouse 26-plex, bead-based Luminex assay (catalogue number EPXR260-26088-901) was used to profile cytokine and chemokine levels in clarified lung supernatants. The assay was performed according to the manufacturer’s instructions, and all incubation steps occurred on an orbital shaker set at 300 r.p.m. Briefly, 50 μl of clarified lung homogenate supernatant was combined with beads in a lidded, black 96-well plate supplied as part of the kit and incubated for 30 min at room temperature, and then overnight at 4 °C. The next day, the plate was allowed to equilibrate to room temperature for 30 min, washed 3 times with 150 μl per well of 1× wash buffer diluted, and then 25 μl per well of 1× detection antibody mixture was added for 30 min at room temperature. The plate was washed 3 times, and then 50 μl per well of 1× Streptavidin–PE solution was added for 30 min at room temperature. After washing 3 times, 120 μl per well of reading buffer was added, and the plate was incubated for 5 min at room temperature. Data were acquired on a Luminex 100/200 analyser (Millipore) with xPONENT software (version 4.3) and analysed using GraphPad Prism (version 8.0) and R (version 4.0.5).

### Micro-CT imaging

Hamsters were inoculated intranasally with 10^3^ PFU (in 30 μl) of B.1.1.529 (strain NC928), B.1.617.2 (UW-5250) or PBS. Lungs of the infected animals were imaged by using an in vivo micro-CT scanner (CosmoScan FX; Rigaku Corporation). Under ketamine–xylazine anaesthesia, the animals were placed in the image chamber and scanned for 2 min at 90 kV, 88 μA, field of view 45 mm and pixel size 90.0 μm. After scanning, the lung images were reconstructed by using the CosmoScan Database software of themicro-CT (Rigaku Corporation) and analysed by using the manufacturer-supplied software. A CT severity score, adapted from a human scoring system, was used to grade the severity of the lung abnormalities^62^. Each lung lobe was analysed for degree of involvement and scored from 0 to 4 depending on the severity: 0 (none, 0%), 1 (minimal, 1%–25%), 2 (mild, 26%–50%), 3 (moderate, 51%–75%) or 4 (severe, 76%–100%). Scores for the five lung lobes were summed to obtain a total severity score of 0–20, reflecting the severity of abnormalities across the three infected groups. Images were anonymized and randomized; the scorer was blinded to the group allocation.

### Pathology

Excised animal tissues were fixed in 4% paraformaldehyde in PBS, and processed for paraffin embedding. The paraffin blocks were cut into 3-µm-thick sections and mounted on silane-coated glass slides. Sections were processed for in situ hybridization using an RNA scope 2.5 HD Red Detection kit (Advanced Cell Diagnostics) with an antisense probe targeting the nucleocapsid gene of SARS-CoV-2 (Advanced Cell Diagnostics). Lung tissue sections were scored for pathology on the basis of the percentage of alveolar inflammation in a given area of a pulmonary section collected from each animal in each group using the following scoring system: 0, no pathological change; 1, affected area (≤10%); 2, affected area (<50%, >10%); 3, affected area (≥50%); an additional point was added when pulmonary edema and/or alveolar haemorrhage was observed.

### Reagent availability

All reagents described in this paper are available through material transfer agreements.

### Statistical analysis

The number of independent experiments and technical replicates used are indicated in the relevant figure legends. Statistical analysis included unpaired *t*-tests, Mann–Whitney tests and ANOVA with multiple correction post tests.

### Reporting summary

Further information on research design is available in the Nature Research Reporting Summary linked to this paper.

## Online content

Any methods, additional references, Nature Research reporting summaries, source data, extended data, supplementary information, acknowledgements, peer review information; details of author contributions and competing interests; and statements of data and code availability are available at 10.1038/s41586-022-04441-6.

## Supplementary information

Supplementary Tables This file contains Supplementary Tables 1–3. Supplementary Table 1: List of experiments and viruses used by different laboratories. Supplementary Table 2: Alignment of key sequence changes in B.1.1.529 Omicron isolates. Supplementary Table 3: Cytokine and chemokine concentration in SARS-CoV-2-infected K18-hACE2 transgenic mice.

Reporting Summary

Supplementary Data This file contains source data for Supplementary Table 3.

## Data Availability

All data supporting the findings of this study are available in the paper. There are no restrictions in obtaining access to primary data. Source data are provided with this paper.

## Source data

Source Data Fig. 1
Source Data Fig. 2
Source Data Fig. 3

## Code availability

No code was used in the course of the data acquisition or analysis.

## References

[1] Callaway E, Ledford H (2021). How bad is Omicron? What scientists know so far. Nature.

[2] Torjesen I (2021). Covid-19: Omicron may be more transmissible than other variants and partly resistant to existing vaccines, scientists fear. BMJ.

[3] Kuiper, M. J. et al. But mouse, you are not alone: on some severe acute respiratory syndrome coronavirus 2 variants infecting mice. *ILAR J.* **12**, ilab031 (2022). 10.1093/ilar/ilab031 PMC9236659 35022734

[4] Wei, C. et al. Evidence for a mouse origin of the SARS-CoV-2 Omicron variant. *J. Genet. Genomics* **48**, 1111–1121 (2021). 10.1016/j.jgg.2021.12.003 PMC8702434 34954396

[5] Muñoz-Fontela C (2020). Animal models for COVID-19. Nature.

[6] Letko M, Marzi A, Munster V (2020). Functional assessment of cell entry and receptor usage for SARS-CoV-2 and other lineage B betacoronaviruses. Nat. Microbiol..

[7] Pinto D (2020). Cross-neutralization of SARS-CoV-2 by a human monoclonal SARS-CoV antibody. Nature.

[8] Cao Y (2020). Potent neutralizing antibodies against SARS-CoV-2 identified by high-throughput single-cell sequencing of convalescent patients’ B cells. Cell.

[9] Zost SJ (2020). Rapid isolation and profiling of a diverse panel of human monoclonal antibodies targeting the SARS-CoV-2 spike protein. Nat. Med..

[10] Barnes CO (2020). SARS-CoV-2 neutralizing antibody structures inform therapeutic strategies. Nature.

[11] Tortorici MA (2020). Ultrapotent human antibodies protect against SARS-CoV-2 challenge via multiple mechanisms. Science.

[12] Rathe JA (2020). SARS-CoV-2 serologic assays in control and unknown populations demonstrate the necessity of virus neutralization testing. J. Infect. Dis..

[13] Wan Y, Shang J, Graham R, Baric RS, Li F (2020). Receptor recognition by novel coronavirus from Wuhan: an analysis based on decade-long structural studies of SARS. J. Virol..

[14] McCray PB (2007). Lethal infection of K18-hACE2 mice infected with severe acute respiratory syndrome coronavirus. J. Virol..

[15] Jiang RD (2020). Pathogenesis of SARS-CoV-2 in transgenic mice expressing human angiotensin-converting enzyme 2. Cell.

[16] Winkler ES (2020). SARS-CoV-2 infection of human ACE2-transgenic mice causes severe lung inflammation and impaired function. Nat. Immunol..

[17] Hassan AO (2020). A SARS-CoV-2 infection model in mice demonstrates protection by neutralizing antibodies. Cell.

[18] Sun J (2020). Generation of a broadly useful model for COVID-19 pathogenesis, vaccination, and treatment. Cell.

[19] Bao L (2020). The pathogenicity of SARS-CoV-2 in hACE2 transgenic mice. Nature.

[20] Sun SH (2020). A mouse model of SARS-CoV-2 infection and pathogenesis. Cell Host Microbe.

[21] Winkler ES (2021). SARS-CoV-2 causes lung infection without severe disease in human ACE2 knock-in mice. J. Virol..

[22] Rathnasinghe, R. et al. The N501Y mutation in SARS-CoV-2 spike leads to morbidity in obese and aged mice and is neutralized by convalescent and post-vaccination human sera. Preprint at 10.1101/2021.01.19.21249592 (2021).

[23] Gu H (2020). Adaptation of SARS-CoV-2 in BALB/c mice for testing vaccine efficacy. Science.

[24] Chen RE (2021). In vivo monoclonal antibody efficacy against SARS-CoV-2 variant strains. Nature.

[25] Kibler, K. V. et al. Intranasal immunization with a vaccinia virus vaccine vector expressing pre-fusion stabilized SARS-CoV-2 spike fully protected mice against lethal challenge with the heavily mutated mouse-adapted SARS2-N501Y_MA30_ strain of SARS-CoV-2. Preprint at 10.1101/2021.12.06.471483 (2021). 10.3390/vaccines10081172 PMC9394475 35893821

[26] Leist SR (2020). A mouse-adapted SARS-CoV-2 induces acute lung injury and mortality in standard laboratory mice. Cell.

[27] Dinnon KH (2020). A mouse-adapted model of SARS-CoV-2 to test COVID-19 countermeasures. Nature.

[28] Wong, L.-Y. R. et al. Eicosanoid signaling as a therapeutic target in middle-aged mice with severe COVID-19. Preprint at 10.1101/2021.04.20.440676 (2021).

[29] Vanderheiden A (2021). CCR2 signaling restricts SARS-CoV-2 infection. mBio.

[30] Muruato A (2021). Mouse-adapted SARS-CoV-2 protects animals from lethal SARS-CoV challenge. PLoS Biol..

[31] Cameroni, E. et al. Broadly neutralizing antibodies overcome SARS-CoV-2 Omicron antigenic shift. *Nature*10.1038/s41586-021-04386-2 (2021). 10.1038/s41586-021-04386-2 PMC9531318 35016195

[32] Sia SF (2020). Pathogenesis and transmission of SARS-CoV-2 in golden hamsters. Nature.

[33] Imai M (2020). Syrian hamsters as a small animal model for SARS-CoV-2 infection and countermeasure development. Proc. Natl Acad. Sci. USA.

[34] Ying B (2021). Protective activity of mRNA vaccines against ancestral and variant SARS-CoV-2 strains. Sci. Transl. Med..

[35] Imai M (2021). Characterization of a new SARS-CoV-2 variant that emerged in Brazil. Proc. Natl Acad. Sci. USA.

[36] Winkler ES (2021). Human neutralizing antibodies against SARS-CoV-2 require intact Fc effector functions for optimal therapeutic protection. Cell.

[37] Simpson S (2020). Radiological Society of North America expert consensus statement on reporting chest CT findings related to COVID-19. Endorsed by the Society of Thoracic Radiology, the American College of Radiology, and RSNA - Secondary Publication. J. Thoracic Imaging.

[38] Damas J (2020). Broad host range of SARS-CoV-2 predicted by comparative and structural analysis of ACE2 in vertebrates. Proc. Natl Acad. Sci. USA.

[39] Gilliland, T. et al. Protection of human ACE2 transgenic Syrian hamsters from SARS CoV-2 variants by human polyclonal IgG from hyper-immunized transchromosomic bovines. Preprint at 10.1101/2021.07.26.453840 (2021).

[40] Espenhain, L. et al. Epidemiological characterisation of the first 785 SARS-CoV-2 Omicron variant cases in Denmark, December 2021. *Euro Surveill*. 10.2807/1560-7917.es.2021.26.50.2101146 (2021). 10.2807/1560-7917.ES.2021.26.50.2101146 PMC8728489 34915977

[41] Kupferschmidt K, Vogel G (2021). How bad is Omicron? Some clues are emerging. Science.

[42] Kenrie, P.Y. et al. SARS-CoV-2 Omicron variant replication in human respiratory tract ex vivo. *Nature*10.1038/s41586-02200447906 (2021).

[43] Abdelnabi, R. et al. The omicron (B.1.1.529) SARS-CoV-2 variant of concern does not readily infect Syrian hamsters. *Antiviral Res.* **198**, 105253 (2022). 10.1016/j.antiviral.2022.105253 PMC8776349 35066015

[44] Yadav P (2021). Isolation of SARS-CoV-2 B.1.1.28.2 (P2) variant and pathogenicity comparison with D614G variant in hamster model. J. Infect. Public Health.

[45] Ulrich, L. et al. Enhanced fitness of SARS-CoV-2 variant of concern Alpha but not Beta. *Nature* **602**, 307–313 (2021). 10.1038/s41586-021-04342-0 PMC8828469 34937050

[46] Griffin BD (2021). SARS-CoV-2 infection and transmission in the North American deer mouse. Nat. Commun..

[47] Zheng J (2021). COVID-19 treatments and pathogenesis including anosmia in K18-hACE2 mice. Nature.

[48] Mannar, D. et al. SARS-CoV-2 Omicron variant: ACE2 binding, cryo-EM structure of spike protein-ACE2 complex and antibody evasion. *Science* **20**, ean7760 (2022). 10.1126/science.abn7760 PMC9799367 35050643

[49] McCallum, M. et al. Structural basis of SARS-CoV-2 Omicron immune evasion and receptor engagement. *Science* **25**, eabn8652 (2022). 10.1126/science.abn8652 PMC9427005 35076256

[50] Zang R (2020). TMPRSS2 and TMPRSS4 promote SARS-CoV-2 infection of human small intestinal enterocytes. Sci. Immunol..

[51] Matsuyama S (2020). Enhanced isolation of SARS-CoV-2 by TMPRSS2-expressing cells. Proc. Natl Acad. Sci. USA.

[52] Chen RE (2021). Resistance of SARS-CoV-2 variants to neutralization by monoclonal and serum-derived polyclonal antibodies. Nat. Med..

[53] Plante, J. A. et al. Spike mutation D614G alters SARS-CoV-2 fitness. *Nature* **592**, 116–121 (2020). 10.1038/s41586-020-2895-3 PMC8158177 33106671

[54] Gagne M (2021). Protection from SARS-CoV-2 Delta one year after mRNA-1273 vaccination in rhesus macaques coincides with anamnestic antibody response in the lung. Cell.

[55] Corbett KS (2021). mRNA-1273 protects against SARS-CoV-2 beta infection in nonhuman primates. Nat. Immunol..

[56] Li Z (2014). Generation of transgenic pigs by cytoplasmic injection of piggyBac transposase-based pmGENIE-3 plasmids. Biol. Reprod..

[57] Case JB, Bailey AL, Kim AS, Chen RE, Diamond MS (2020). Growth, detection, quantification, and inactivation of SARS-CoV-2. Virology.

[58] Vanderheiden, A. et al. CCR2 signaling restricts SARS-CoV-2 infection. *mBio* **12**, e0274921 (2021). 10.1128/mBio.02749-21 PMC8576528 34749524

[59] Jangra S (2021). Sterilizing immunity against SARS-CoV-2 infection in mice by a single-shot and lipid amphiphile imidazoquinoline TLR7/8 agonist-adjuvanted recombinant spike protein vaccine. Angew. Chem. Int. Ed..

[60] Chu DKW (2020). Molecular diagnosis of a novel coronavirus (2019-nCoV) causing an outbreak of pneumonia. Clin. Chem..

[61] Halfmann P (2021). SARS-CoV-2 interference of influenza virus replication in Syrian hamsters. J. Infect. Dis..

[62] Chung M (2020). CT imaging features of 2019 novel coronavirus (2019-nCoV). Radiology.

