# Supplementary material for: SARS-CoV-2 Omicron virus causes attenuated disease in mice and hamsters
Source: Nature. 2022 Jan 21;603(7902):687–92. doi: 10.1038/s41586-022-04441-6 (PMC8942849; doi:10.1038/s41586-022-04441-6)
Supplement: Supplementary file 1 — This file contains Supplementary Tables 1–3. Supplementary Table 1: List of experiments and viruses used by different laboratories. Supplementary Table 2: Alignment of key sequence changes in B.1.1.529 Omicron isolates. Supplementary Table 3: Cytokine and chemokine concentration in SARS-CoV-2-infected K18-hACE2 transgenic mice. [file 41586_2022_4441_MOESM1_ESM.pdf]

nature portfolio

https://doi.org/10.1038/s41586-022-04441-6

Supplementary information

SARS-CoV-2 Omicron virus causes attenuated disease in mice and hamsters

In the format provided by the authors and unedited

Supplemental Table 1. List of experiments and viruses used by different laboratories

Laboratory	Animal model	Phenotype	Virus detection method	Days post infection	Virus isolates
Kawaoka (YK)	BALB/c mice	Virus titer in Nasal turbinates and Lungs; WBP*	Infectious virus titer	2	B.1.351 (hCoV-19/USA/MD-HP01542/2021)<br>B.1.1.529 (hCoV-19/Japan/NC928-2N/2021)
	Syrian Hamster	Weight loss; Virus titer in Nasal turbinates and Lungs; WBP; CT scan[&]	Infectious virus titer	3	B.1.617.2 (hCoV-19/USA/WI-UW-5250/2021)<br>B.1.1.529 (hCoV-19/Japan/NC928-2N/2021)
	hACE2-transgenic Syrian Hamster	Weight loss; Survival; Virus titer in Nasal turbinates and Lungs	Infectious virus titer	3 and 5	HP-095 (SARS-CoV-2/UT-HP095-1N/Human/2020/Tokyo)<br>B.1.1.529 (hCoV-19/USA/WI-WSLH-221686/2021)
Suzuki (TS)	Syrian Hamster	Histopathology			B.1.617.2 (hCoV-19/USA/WI-UW-5250/2021)<br>B.1.1.529 (hCoV-19/Japan/NC928-2N/2021)
Diamond (MD)	K18-hACE2 transgenic mice	Weight loss; Virus titer in Lungs	RT-qPCR and Infectious virus titer	3	WA1/2020-D614G<br>B.1.1.529 (hCoV-19/USA/WI-WSLH-221686/2021)
Boon (AB)	Syrian Hamster	Weight loss; Virus titer in Nasal wash and Lungs	RT-qPCR	4	WA1/2020-D614G<br>B.1.1.529 (hCoV-19/USA/WI-WSLH-221686/2021)
Seder (RS)	Syrian Hamster	Weight loss	-	-	B.1.1.529 (hCoV-19/USA/GA-EHC-2811C/2021)<br>B.1.617.2 (hCoV-19/USA/PHC658/202)
Garcia-Sastre (AGS)	129 mice	Weight loss; Virus titer in Nasal turbinates and Lungs	Infectious virus titer	4	B.1.1.529 (hCoV-19/USA/NY-MSHSPSP-PV44476/2021)<br>B.1.1.529 (hCoV-19/USA/NY-MSHSPSP-PV44488/2021)<br>B.1.351 (hCoV-19/USA/MD-HP01542/2021)

	K18-hACE2 transgenic mice	Weight loss; Virus titer in Nasal turbinates and Lungs; Cytokine production	Infectious virus titer	3	B.1.1.529 (hCoV-19/USA/NY-MSHSPSP-PV44476/2021)<br>B.1.351 (hCoV-19/USA/MD-HP01542/2021)
Perlman (SP)	C57BL/6 mice	Weight loss	-	-	B.1.1.529 (hCoV-19/USA/GA-EHC-2811C/2021)<br>B.1.351 (hCoV19/20H/501Y.V2)<br>B.1.1.7 (hCoV-19/USA/CA_CDC_5574/2020)
Suthar (MS)	129 mice	Weight loss; Virus titer in Nasal turbinates and Lungs	RT-qPCR	3	B.1.1.529 (hCoV-19/USA/GA-EHC-2811C/2021)<br>B.1.351 (hCoV-19/USA/MD-HP01542/2021)
Webby (RW)	Syrian hamster	Weight loss; Virus titer in Nasal wash	RT-qPCR	4	B.1.1.529 (hCoV-19/USA/GA-EHC-2811C/2021)<br>USA-WA1/2020

* Whole Body Plethysmography, & Computerized tomography scan

Supplementary Table 2. Alignment of Key Sequence Changes in B.1.1.529 Omicron Isolates

Gene	NT change	AA change[a]	7263803[b]	7171744[b]	7507055[b]	7908052[b]	7908059[b]
ORF1ab	A2832G	K856R					
ORF1ab	6513_6515 del	SL2083I					
ORF1ab	G8393A	A2710T					
ORF1ab	C10029T	T3255I					
ORF1ab	C10449A	P3395H					
ORF1ab	11288_11296 del	SGF3675 del					
ORF1ab	C14408T	P4715L					
ORF1ab	A18163G	I5967V					
ORF1ab	C21034T	L6924F					
S	C21762T	A67V					
S	21765_21770 del	HV69 del					
S	C21846T	T95I					
S	21987_21995 del	G142D					
S	21987_21995 del	VYY143 del					
S	22194_22196 del	NL211I					
S	22205GAGCCAGAA ins	214EPE ins					
S	G22578A	G339D					
S	C22674T	S371F					
S	T22679C	S373P					
S	C22686T	S375F					
S	G22813T	K417N					
S	T22882G	N440K					
S	G22898A	G446S					
S	G22992A	S477N					
S	C22995A	T478K					
S	A23013C	E484A					
S	A23040G	Q493R					
S	G23048A	G496S					
S	A23055G	Q498R					
S	A23063T	N501Y					
S	T23075C	Y505H					
S	C23202A	T547K					
S	A23403G	D614G					
S	C23525T	H655Y					

S	T23599G	N679K					
S	C23604A	P681H					
S	C23632T	A701V					
S	C23854A	N764K					
S	G23948T	D796Y					
S	C24130A	N856K					
S	A24424T	Q954H					
S	T24469A	N969K					
S	C24503T	L981F					
E	C26270T	T9I					
M	A26530G	D3G					
M	C26577G	Q19E					
M	G26709A	A63T					
N	C28311T	P13L					
N	28362_28370 del	ERS31 del					
N	G28881A, G28882A, G28883C	RG203KR					

[a]Green cells indicate the presence of indicated amino acid substitution in a given isolate; white cells indicate the absence of the substitution in a given isolate.
[b] Isolate identifiers correspond to GISAID sequences

Supplemental Table 3: Cytokine and chemokine concentration in SARS-CoV-2-infected K18-hACE2 transgenic mice

Cytokine/Chemokine	Concentration (mean ± SD (pg/mL))		
	**Mock**	**B.1.351**	**B.1.1.529 + A701V**
IL-10	2.7 ± 0.9	2.0 ± 0.0	3.2 ± 1.5
IL-1β	1.4 ± 0.0	2.3 ± 1.0	1.6 ± 0.2
IL-2	7.8 ± 1.8	4.7 ± 2.6	7.1 ± 5.5
CXCL10	17.8 ± 0.7	155.4 ± 4.7	42.8 ± 28.1
IL-4	1.4 ± 0.0	1.4 ± 0.0	1.4 ± 0.0
IL-5	2.2 ± 0.0	5.4 ± 2.3	5.2 ± 4.5
IL-6	6.7 ± 1.7	452.2 ± 357.8	12.5 ± 7.5
IL-22	13.0 ± 0.0	13.6 ± 1.1	14.2 ± 2.1
IL-9	38.7 ± 15.4	34.3 ± 14.6	56.9 ± 32.0
IL-13	2.9 ± 0.0	2.9 ± 0.0	3.2 ± 0.6
IL-27	2.8 ± 0.2	2.7 ± 0.1	2.6 ± 0.0
IL-23	19.3 ± 6.3	26.8 ± 13.3	37.1 ± 28.2
IFN-γ	2.2 ± 0.0	4.9 ± 2.8	2.2 ± 0.0
IL-12p70	2.3 ± 0.0	2.8 ± 0.5	2.3 ± 0.0
GM-CSF	3.2 ± 0.0	8.0 ± 1.4	3.4 ± 0.4
CXCL1	20.1 ± 1.8	136.8 ± 80.6	25.0 ± 11.5
CCL5	441.3 ± 98.9	453.1 ± 226.1	268.5 ± 165.9
TNF-α	3.7 ± 0.0	14.9 ± 12.0	5.1 ± 2.0
CCL3	8.2 ± 1.2	59.6 ± 7.4	10.4 ± 4.1
CCL7	34.0 ± 26.5	790.0 ± 363.8	57.1 ± 42.7
CCL2	77.8 ± 9.8	535.3 ± 343.0	91.9 ± 47.4
IL-17A	156.3 ± 0.0	156.3 ± 0.0	172.4 ± 28.0
CXCL2	9.5 ± 0.1	27.9 ± 3.4	13.8 ± 4.5
CCL11	175.6 ± 29.6	212.9 ± 75.6	387.7 ± 306.3
IL-18	78.2 ± 34.4	220.2 ± 124.1	89.1 ± 50.6
CCL4	14.9 ± 1.5	34.0 ± 15.4	9.9 ± 3.9
