# Supplementary material for: SARS-CoV-2 Omicron virus causes attenuated disease in mice and hamsters
Source: Nature. 2022 Jan 21;603(7902):687–92. doi: 10.1038/s41586-022-04441-6 (PMC8942849; doi:10.1038/s41586-022-04441-6)
Supplement: Supplementary file 2 — Reporting Summary [file 41586_2022_4441_MOESM2_ESM.pdf]

nature research

Corresponding author(s): Michael Diamomd

Last updated by author(s): 01/14/2022

Reporting Summary

Nature Research wishes to improve the reproducibility of the work that we publish. This form provides structure for consistency and transparency in reporting. For further information on Nature Research policies, see our Editorial Policies and the Editorial Policy Checklist.

Statistics

For all statistical analyses, confirm that the following items are present in the figure legend, table legend, main text, or Methods section.

n/a	Confirmed	
☐	☒	The exact sample size (*n*) for each experimental group/condition, given as a discrete number and unit of measurement
☐	☒	A statement on whether measurements were taken from distinct samples or whether the same sample was measured repeatedly
☐	☒	The statistical test(s) used AND whether they are one- or two-sided<br>*Only common tests should be described solely by name; describe more complex techniques in the Methods section.*
☒	☐	A description of all covariates tested
☒	☐	A description of any assumptions or corrections, such as tests of normality and adjustment for multiple comparisons
☐	☒	A full description of the statistical parameters including central tendency (e.g. means) or other basic estimates (e.g. regression coefficient) AND variation (e.g. standard deviation) or associated estimates of uncertainty (e.g. confidence intervals)
☐	☒	For null hypothesis testing, the test statistic (e.g. *F*, *t*, *r*) with confidence intervals, effect sizes, degrees of freedom and *P* value noted<br>*Give P values as exact values whenever suitable.*
☒	☐	For Bayesian analysis, information on the choice of priors and Markov chain Monte Carlo settings
☒	☐	For hierarchical and complex designs, identification of the appropriate level for tests and full reporting of outcomes
☒	☐	Estimates of effect sizes (e.g. Cohen's *d*, Pearson's *r*), indicating how they were calculated

Our web collection on statistics for biologists contains articles on many of the points above.

Software and code

Policy information about availability of computer code

Data collection	No software was used in this study to collect data
Data analysis	Prism 8.0 was used to perform all statistical analysis. xPONENT (version 4.3) and R (version 4.0.5) were used to analyze the cytokine data.

For manuscripts utilizing custom algorithms or software that are central to the research but not yet described in published literature, software must be made available to editors and reviewers. We strongly encourage code deposition in a community repository (e.g. GitHub). See the Nature Research guidelines for submitting code & software for further information.

Data

Policy information about availability of data

All manuscripts must include a data availability statement. This statement should provide the following information, where applicable:

- Accession codes, unique identifiers, or web links for publicly available datasets
- A list of figures that have associated raw data
- A description of any restrictions on data availability

All data supporting the findings of this study are available within the paper and in the Source Data. There are no restrictions in obtaining access to primary data.

Field-specific reporting

Please select the one below that is the best fit for your research. If you are not sure, read the appropriate sections before making your selection.

☒ Life sciences ☐ Behavioural & social sciences ☐ Ecological, evolutionary & environmental sciences

For a reference copy of the document with all sections, see nature.com/documents/nr-reporting-summary-flat.pdf

Life sciences study design

All studies must disclose on these points even when the disclosure is negative.

Sample size	No sample sizes were chosen a priori. All experiments with statistical analysis were repeated at least two independent times, each with multiple technical replicates. Experimental size of cohorts was determined based on prior experience performing studies in animals and with SARS-CoV-2 infection models (PMID: 34846168, 34668780, 32839612, 34140350, and 32571934). The studies were corroborated by independent and parallel experiments by several different groups in the SAVE/NIAID consortia
Data exclusions	No data was excluded.
Replication	All experiments with multiple biological and/or technical replicates are indicated the Figure legends.
Randomization	For animal studies, mice were randomly assigned to treatment groups in an age and sex-matched distribution. All experiments were derived from animal work so no additional randomization was required in downstream analysis.
Blinding	No blinding was performed although several key studies were performed independently by different members of the SAVE consortium. Blinding was not performed because studies were independently corroborated by separate laboratories.

Reporting for specific materials, systems and methods

We require information from authors about some types of materials, experimental systems and methods used in many studies. Here, indicate whether each material, system or method listed is relevant to your study. If you are not sure if a list item applies to your research, read the appropriate section before selecting a response.

Materials & experimental systems

n/a	Involved in the study	
☒	☐	Antibodies
☐	☒	Eukaryotic cell lines
☒	☐	Palaeontology and archaeology
☐	☒	Animals and other organisms
☒	☐	Human research participants
☒	☐	Clinical data
☒	☐	Dual use research of concern

Methods

n/a	Involved in the study	
☒	☐	ChIP-seq
☒	☐	Flow cytometry
☒	☐	MRI-based neuroimaging

Eukaryotic cell lines

Policy information about cell lines

Cell line source(s)	Vero-TMPRSS2, Diamond and Kawaoka laboratories (not commercially available); Vero-hACE2-TMPRSS2, Graham laboratory, VRC/NIH (available at BEI Resources, NR-54970)
Authentication	These were obtained from academic laboratories and grew and performed as expected. Cells expressing TMPRSS2 and hACE2 were validated using monoclonal antibodies and flow cytometry.
Mycoplasma contamination	All cell lines are routinely tested each month and were negative for mycoplasma.
Commonly misidentified lines (See ICLAC register)	This study did not involve any commonly misidentified cell lines.

Animals and other organisms

Policy information about studies involving animals; ARRIVE guidelines recommended for reporting animal research

Laboratory animals	Heterozygous female K18-hACE2 C57BL/6J mice (strain 2B6.Cg-Tg(K18-ACE2)2Prlmn/J, 5-6 month-old), Male and female 129 mice (strain: 129S2/SvPasCrl or 129S1/SvImJ, 6-8, 10-20, or 31 week-old)), and female C57BL/6 (strain 000664, 10-14 month-old) mice were obtained from The Jackson Laboratory and Charles River Laboratories. Female BALB/c mice (6 week-old) were purchased from

Japan SLC Inc.

Five-to-six-week-old male Syrian Golden hamsters were obtained from Charles River Laboratories, Envigo, or Japan SLC Inc. The K18-hACE2 transgenic hamster line will be described in detail elsewhere (see REF 39; bioRxiv, doi:10.1101/2021.07.26.453840),

Mice were housed in groups of 4 to 5; Hamsters were housed alone. Photoperiod = 12 hr on:12 hr off dark/light cycle. Ambient animal room temperature is 70° F, controlled within ±2° and room humidity is 50%, controlled within ±5%.

Wild animals	No wild animals were used in this study.
Field-collected samples	No field collected samples were used in this study.
Ethics oversight	Animal studies were carried out in accordance with the recommendations in the Guide for the Care and Use of Laboratory Animals of the National Institutes of Health. The protocols were approved by the Institutional Animal Care and Use Committee at the Washington University School of Medicine (assurance number A3381–01), University of Wisconsin, Madison (V006426), St. Jude Children's Research Hospital (Assurance number D16-00043), Emory University, University of Iowa (assurance number A3021-01), Icahn School of Medicine at Mount Sinai (PROTO202100007), BIOQUAL, Inc., and the Animal Experiment Committee of the Institute of Medical Science, the University of Tokyo (approval numbers PA19-72 and PA19-75).

Note that full information on the approval of the study protocol must also be provided in the manuscript.
